# Composite Measures of Individual and Area-Level Socio-Economic Status Are Associated with Visual Impairment in Singapore

**DOI:** 10.1371/journal.pone.0142302

**Published:** 2015-11-10

**Authors:** Win Wah, Arul Earnest, Charumathi Sabanayagam, Ching-Yu Cheng, Marcus Eng Hock Ong, Tien Y. Wong, Ecosse L. Lamoureux

**Affiliations:** 1 Centre for Infectious Disease Epidemiology and Research, Saw Swee Hock School of Public Health, National University of Singapore, Singapore, Singapore; 2 Duke-NUS Graduate Medical School, Singapore, Singapore; 3 Department of Epidemiology and Preventive Medicine, School of Public Health and Preventive Medicine, Monash University, Melbourne, Australia; 4 Singapore Eye Research Institute, Singapore National Eye Centre, Singapore, Singapore; 5 Department of Ophthalmology, National University of Singapore and National University Health System, Singapore, Singapore; 6 Department of Emergency Medicine, Singapore General Hospital, Singapore, Singapore; 7 Centre for Eye Research Australia, Royal Victorian Eye and Ear Hospital, University of Melbourne, Melbourne, Australia; Swinburne University of Technology, AUSTRALIA

## Abstract

**Purpose:**

To investigate the independent relationship of individual- and area-level socio-economic status (SES) with the presence and severity of visual impairment (VI) in an Asian population.

**Methods:**

Cross-sectional data from 9993 Chinese, Malay and Indian adults aged 40–80 years who participated in the Singapore Epidemiology of eye Diseases (2004–2011) in Singapore. Based on the presenting visual acuity (PVA) in the better-seeing eye, VI was categorized into normal vision (logMAR≤0.30), low vision (logMAR>0.30<1.00), and blindness (logMAR≥1.00). Any VI was defined as low vision/blindness in the PVA of better-seeing eye. Individual-level low-SES was defined as a composite of primary-level education, monthly income<2000 SGD and residing in 1 or 2-room public apartment. An area-level SES was assessed using a socio-economic disadvantage index (SEDI), created using 12 variables from the 2010 Singapore census. A high SEDI score indicates a relatively poor SES. Associations between SES measures and presence and severity of VI were examined using multi-level, mixed-effects logistic and multinomial regression models.

**Results:**

The age-adjusted prevalence of any VI was 19.62% (low vision = 19%, blindness = 0.62%). Both individual- and area-level SES were positively associated with any VI and low vision after adjusting for confounders. The odds ratio (95% confidence interval) of any VI was 2.11(1.88–2.37) for low-SES and 1.07(1.02–1.13) per 1 standard deviation increase in SEDI. When stratified by unilateral/bilateral categories, while low SES showed significant associations with all categories, SEDI showed a significant association with bilateral low vision only. The association between low SES and any VI remained significant among all age, gender and ethnic sub-groups. Although a consistent positive association was observed between area-level SEDI and any VI, the associations were significant among participants aged 40–65 years and male.

**Conclusion:**

In this community-based sample of Asian adults, both individual- and area-level SES were independently associated with the presence and severity of VI.

## Introduction

Individual- and area-level measures of socio-economic status (SES) are independent factors influencing major diseases and health outcomes[1,2]. In many developed countries, composite measures of SES and socio-economic deprivation such as SEIFA (Socio-Economic Indices for Australia) in Australia, and Carstairs index in United Kingdom have been created[3,4]. Such indices are useful for geographically targeted resource allocation, research and health education/interventions, and can be used to determine funding formula for primary healthcare services, social services, relating SES with health outcomes and risk factors/behaviours, as well as aid community-based service providers in terms of pricing and pitching the appropriate services for communities with different SES.

Visual impairment (VI) is a worldwide problem with huge socio-economic consequences[5]. Individual low SES measured as low income, education or social class has been shown to be associated with VI in several studies[6]. At a population level, distribution of VI may be related to socio-economic factors[6]. This is particularly true in Asia where there is rising income inequality in many newly developed countries, such as China, Taiwan, Singapore[7]. Both individual and areal level SES were reported to have independent predictive power in capturing community wide health disparities[8]. In Singapore, we have previously reported an association between VI and individual- and area-level measures of SES such as low income, education, and occupation among Indians and Malays[9,10]. No study to date however has looked at the relationship of a composite socio-economic disadvantage index (SEDI) which includes several socio-economic measures with the presence and severity of VI in Singapore.

We recently created a socio-economic disadvantage index (SEDI) to measure area-level SES that reflects the composite socio-economic circumstances (household and personal income, housing, education, occupation)[11]. A single composite index would be more meaningful in understanding areal level factors which allows comparisons between groups and useful for geographically targeted resource allocation, research and health education/interventions for communities with different SES.

The aim of the current study was to investigate the independent association of individual and area-level SES parameters with the presence and severity of VI in a large and multi-ethnic Asian population in Singapore using the individual level SES and the recently created SEDI score representing area-level SES[11].

## Materials and Methods

### Study population and setting

Singapore is an island state with a total land area of 700km^2[^[12]. Based on the latest census data, Singapore’s total population was 5.08 million as at end-June 2010, of which 3.77 million were Singapore residents[12]. The three major ethnic groups in Singapore are Chinese, Malay and Indian with the majority of migrants from across Asia. Most Chinese in Singapore are ethnic descendants of immigrants from the outlying provinces of china (Fujian and Guangdong) with several different dialect groups consisting of Hokkien (41%), Teochew (21%), and Hainanese (5%), Cantonese (15%), Hakka (11.4%) and other minority groups[13,14]. Singapore Indian residents encompass persons with ancestry originating from the Indian subcontinent, including India, Pakistan, Bangladesh, Sri Lanka and Nepal[13,14]. Singapore’s Malay residents include all people of Malay or Indonesian origin (e.g., Javanese, Boyanese, and Bugis)[15].^15^

### Individual-level SES and covariates data

Data on individual-level SES, covariates and VI outcomes were derived from the Singapore Epidemiology of Eye Diseases (SEED) Program comprising of population-based cross-sectional data including the three major ethnic groups (Chinese, Malays and Indians) in Singapore: The Singapore Malay Eye Study (SiMES, 2004–2006), the Singapore Indian Eye Study (SINDI, 2007–2009), and the Singapore Chinese Eye Study (SCES, 2009–2011). These studies followed the same study design and sampling areas as previously published[13,15]. They used age-stratified random sampling to select participants in each ethnic group and recruited 3280 ethnic Malays, 3400 Indians, and 3353 Chinese aged 40–80 years residing in the South-Western part of Singapore, including 8 development guide plan (DGP) areas (Bukit Batok, Bukit Merah, Bukit Timah, Clementi, Jurong East, Jurong West, Outram and Queenstown). Sampling areas of these studies were chosen in the south-western part of Singapore due to a fair representation of Singapore resident population in terms of age distribution, housing types and socio-economic status[11,12,16].^,^ Written, informed consent was obtained from each participant in both studies and the studies adhered to the Declaration of Helsinki. Ethical approval was obtained from the Institutional Review Board at the Singapore Eye Research Institute.

Education status, monthly income and housing status were used as measures of individual-level SES. Information on these SES measures was obtained using a standardized questionnaire. Persons were classified by educational level into three categories: 1) primary or lower (≤6 years), 2) secondary (7 to 10 years) and 3) post-secondary (≥11 years, including university education). Income was based in Singapore dollars (SGD) and three income categories were created: 1) low (≤1000), 2) middle (1001-$2000), and 3) high (>2000). Housing type was classified as follows: 1) small size public apartments (1–2 rooms), 2) medium size public apartments (3 rooms), and 3) large public apartments (>4 rooms) or private housing. We created a composite ‘low SES’ variable defined as primary or below education, monthly income less than 2000 SGD and residing in 1 to 2-room apartments[17].

Information on covariates were obtained from a standardized interview questionnaire (demographic, life-style, medication and medical history), physical (anthropometric and blood pressure) and laboratory examination (blood glucose, and lipid profile). Diabetes mellitus was defined as random blood glucose of ≥ 11.1 mmol/l, use of diabetic medication or a physician diagnosis of diabetes[11,18].^,^ Hypertension was defined as having a systolic blood pressure ≥ 140 mmHg and diastolic BP ≥ 90 mmHg, or the use of anti-hypertensive drugs[11,19].^,^ Hyperlipidaemia was defined as total cholesterol ≥ 6.2 mmol/l or the use of lipid lowering medications[11,20].^,^ Cardiovascular disease (CVD) history was defined as a self-reported history of angina, heart attack or stroke[21]. Smoking was categorized into current, past and never smoker and alcohol drinking was categorized into drinkers and non-drinkers.

### Area-level SES data

An area-level SES was assessed using a SEDI created using 12 variables from the 2010 Singapore census through a principal component analysis[12,22] Details of the process derived socio-economic indices were mentioned in the previous study[11]. Out of initial 23 area attributes from the census, the following 12 area attributes were included; primary education and below; not literate; unemployed; construction industry; hotels and restaurants industry; clerical workers; service and sales workers; plant & machine operators & assemblers; cleaners; laborers & related workers; monthly personal income less than SGD 2,500; monthly household income < SGD 4000, A high SEDI score indicates a relatively poor SES.

### Assessment of outcomes

Visual acuity (VA) scores were measured by logarithm of the minimum angle of resolution (log MAR) charts[23]. VI was defined based on presenting VA (PVA) to take into account VI due to uncorrected refractive error which could reflect low SES. Based on PVA in the better-seeing eye, presence and severity of VI was categorized into no VI (PVA 20/40 or better, logMAR≤0.30), low vision (PVA worse than 20/40 but better than 20/200, logMAR>0.30-<1.00], blindness (PVA of 20/200 or worse], logMAR ≥1.00][24–26]. Any VI was defined as low vision/blindness in the PVA of better-seeing eye. In addition to defining VI based on the better-seeing eye, we also defined VI based on PVA in the worse seeing eye into six mutually exclusive categories: bilateral normal vision (reference), unilateral low and normal vision, bilateral low vision, unilateral blindness and normal vision, unilateral blindness and low vision and bilateral blindness[27].

### Statistical Analyses

Data analysis was performed using Stata version 13.0 (Stata Corp, College Station, Tx, USA) and level of significance was set at *p*<0.05. We combined all three ethnic groups for the main analysis (n = 10033). Age-adjusted prevalence rates of VI and blindness were calculated by the direct method using the year 2010 Singapore census population as the standard population[12]. We used a multi-level mixed-effects logistic regression to identify an independent association between individual-level SES and area-level SEDI and the presence of any VI by taking into account the clustering of individuals within DGP areas[28]. Generalized linear latent and mixed models (GLLAMM) package was used to analyse different multi-level mixed effects models for the multinomial outcomes of presence and severity of VI, low vision and blindness[29,30]. Statistical assessment of interaction between individual- and areal-level low SES was performed by fitting models containing cross-product terms. Associations were examined after adjusting for individual demographic (age, gender, ethnicity), medical (hypertension, diabetes, hyperlipidaemia, history of cardiovascular disease [CVD]) and life-style (alcohol and smoking status) risk factors. Finally, we performed sub-group analyses stratified by age groups (40–65, 65–74 and ≥75 years), gender (male, female) and ethnic (Chinese, Malay, Indian) groups. We also examined the association of low SES and SEDI with characteristics of participants by multivariate logistic and linear regression models.

## Results

Out of 10,033 participants, 9993 (99.6%) were included for the final analysis after excluding those with unknown outcomes and DGP areas. The crude and age-adjusted prevalence of any VI were 27.96% (95% confidence interval [CI], 27.08–28.84%) and 19.62% (18.8–20.4%), respectively and that of low vision and blindness were 19.00% (18.18–19.82%) and 0.62% (0.47–0.77%) respectively. VI data were assigned to 8 DGP areas only since the sampling area of SiMES, SINDI, and SCES was located in the South-Western part of Singapore. SEDI scores of the included DGP areas ranged from 79.8 to 120.1, Bukit Batok (100.6), Bukit Merah (110.1), Bukit Timah (79.8), Clementi (100.6), Jurong East (99.9), Jurong West (101.6), Outram (120.1), and Queenstown (106.9) **(Table 1)**. Compared to participants with normal vision, those with low vision and blindness were more likely to be older, female, Malays, had lower SES and higher prevalence of smoking, diabetes, hypertension, hyperlipidaemia, and CVD. Under corrective refractive error accounted for the majority of any VI (54.9%) and low vision (48.5%) and cataract represented a large proportion of blindness (61.5%) (data not shown). The association of both individual and area-level SES with selected participants’ characteristics is shown in **Table 2**. Low individual SES was associated with older age, female, Malay and Indian ethnicity, current and past smoking, diabetes, hypertension, CVD, and higher SEDI scores. Ever consumption of alcohol was inversely associated with low SES. Increasing age, diabetes mellitus, Malay, Indian, and low SES were associated with higher SEDI scores.

**Table 1:** Characteristics of participants in the Singapore Epidemiology of Eye Diseases study stratified by the severity of visual impairment.

Characteristics	Normal vision n = 7199 (%)	Low vision n = 2685 (%)	Blindness n = 109 (%)	p value†
**Age, years**				
40–49	2169 (30.13)	287 (10.69)	9 (8.26)	<0.001
50–59	2517 (34.96)	605 (22.53)	14 (12.84)	
60–69	1697 (23.57)	830 (30.91)	28 (25.69)	
70–79	816 (11.33)	963 (35.87)	58 (53.21)	
**Sex**				
Male	3691 (51.27)	1200 (44.69)	34 (31.19)	<0.001
Female	3508 (48.73)	1485 (55.31)	75 (68.81)	
**Diabetes mellitus**	1554 (21.59)	783 (29.16)	39 (35.78)	<0.001
**Hypertension**	4097 (56.91)	1951 (72.66)	94 (86.24)	<0.001
**Hyperlipidemia**	3020 (41.95)	1315 (48.98)	59 (54.13)	<0.001
**Cardiovascular disease**	133 (1.85)	113 (4.21)	9 (8.26)	<0.001
**Low socio-economic status**	3427 (47.6)	2006 (74.71)	91 (83.49)	<0.001
**Smoking status**				
Never	4999 (69.44)	1858 (69.2)	82 (75.23)	<0.001
Past	994 (13.81)	430 (16.01)	19 (17.43)	
Current	1200 (16.67)	390 (14.53)	6 (5.5)	
**Ever alcohol drinker**	664 (9.22)	182 (6.78)	1 (0.92)	<0.001
**DGP(SEDI score)**				<0.001
Bukit Batok (100.6)	1114 (15.47)	382 (14.23)	17 (15.6)	
Bukit Merah (110.1)	1173 (16.29)	595 (22.16)	19 (17.43)	
Bukit Timah (79.8)	156 (2.17)	37 (1.38)	0 (0)	
Clementi (100.6)	984 (13.67)	417 (15.53)	9 (8.26)	
Jurong East (99.9)	964 (13.39)	284 (10.58)	19 (17.43)	
Jurong West (101.6)	2371 (32.94)	774 (28.83)	29 (26.61)	
Outram (120.1)	47 (0.65)	28 (1.04)	3 (2.75)	
Queenstown (106.9)	390 (5.42)	168 (6.26)	13 (11.93)	

DGP, Development guide plan; SEDI, Socio-economic Disadvantage Index; SES, Socio-economic status

†p-value represents the difference in characteristics by severity of vision impairment based on chi-square test.

**Table 2:** Association of low SES and SEDI with characteristics of participants.

Variables	Individual level Low SES OR (95% CI)†	Area-level SEDI score Coefficients (95% CI)†
*Individual level*		
**Age, years**		
40–49	Reference	Reference
50–59	1.55 (1.37–1.75)*	-0.11 (-0.39–0.17)
60–69	3.79 (3.3–4.35)*	0.44 (0.13–0.76)*
70–79	9.75 (8.16–11.64)*	0.88 (0.52–1.24)*
**Sex (Female)**	3.16 (2.81–3.56)*	0.11 (-0.14–0.37)
**Diabetes Mellitus**	1.21 (1.08–1.36)*	0.39 (0.13–0.64)*
**Hypertension**	1.49 (1.35–1.65)*	-0.02 (-0.25–0.21)
**Dyslipidemia**	0.95 (0.86–1.05)	-0.07 (-0.28–0.15)
**Cardiovascular disease**	1.71 (1.23–2.38)*	0.12 (-0.54–0.77)
**Smoking**		
Never smoker	Reference	Reference
Current smoker	2.23 (1.92–2.6)*	0.27 (-0.06–0.61)
Past smoker	1.29 (1.1–1.51)*	-0.26 (-0.6–0.08)
**Ever alcohol drinker**	0.74 (0.62–0.88)*	-0.15 (-0.54–0.24)
**Ethnicity**		
Chinese	Reference	Reference
Malay	2.56 (2.27–2.89)*	1.2 (0.94–1.46)*
Indian	1.28 (1.14–1.43)*	0.5 (0.24–0.76)*
**Low socio-economic statu** *s*		0.64 (0.41–0.87)*
*Areal level*		
**SEDI**	1.03 (1.02–1.04)*	

OR, Odds ratio; CI, Confidence interval

*(p<0.05)

†Adjusted for age, sex, ethnicity, diabetes, hypertension, dyslipidemia, cardiovascular disease, and alcohol and smoking status, SEDI; Socio-economic Disadvantage Index.

**Table 3** shows the associations of both individual and area-level SES with the presence and severity of VI. Individual low SES was associated with the presence of any VI, low vision and blindness. Area-level SEDI score was positively associated with the presence of any VI and low vision. The odds ratio/OR (95% CI) of any VI was 2.11(1.88–2.37) for low SES and 1.07(1.02–1.13) per 1 standard deviation increase in SEDI. When stratified by unilateral/bilateral categories, low SES showed significant associations with all severity categories, in particular with bilateral blindness (OR = 2.97, 95% CI = 1.60–5.47) and unilateral blindness and low vision (OR [95% CI] = 3.82 [2.69–5.37]). SEDI showed a significant association with bilateral low vision only (1.09, 1.02–1.15 per 1 SD increase in SEDI). There was a significant interaction between individual and areal level SES for the presence of any VI, low vision and blindness and all severity categories. In sub-group analyses, the association between individual low SES and any VI remained significant among all age, gender and ethnic groups and majority of the DGP areas (**Table 4**). Although a consistent positive association was observed between area-level SEDI and any VI, the associations were significant among participants aged between 40 and 65 years, male and individual low SES. The results from interaction and sub-group analyses showed that the effect of areal level SEDI on VI differed with individual SES and the effect of individual low SES on VI differed in geographic areas.

**Table 3:** Multi-level analysis of individual and area-level SES with the presence and severity of visual impairment.

VI categories	Individual level low SES OR (95% CI)†	Area-level SEDI score‡ OR (95% CI)†	Interaction^ p value†
**Presence of any VI (better-seeing eye)**	2.11 (1.88–2.37)*	1.07 (1.02–1.13)*	<0.001*
**Severity of VI (better-seeing eye)**			
No vision impairment	Reference		
Low vision	2.1 (1.87–2.35)*	1.07 (1.02–1.13)*	<0.001*
Blindness	2.53 (1.37–4.69)*	1.1 (0.89–1.35)	0.002*
**Unilateral/bilateral low vision/blindness (worse-seeing eye)**			
Bilateral Normal vision	Reference			
Unilateral low vision and normal vision	1.42 (1.26–1.6)*	1.03 (0.98–1.09)	0.001*
Bilateral low vision	2.27 (1.99–2.61)*	1.09 (1.02–1.15)*	<0.001*
Unilateral blindness and normal vision	1.58 (1.14–2.23)*	1.09 (0.94–1.26)	<0.001*
Unilateral blindness and low vision	3.82 (2.69–5.37)*	1.07 (0.95–1.21)	0.005*
Bilateral blindness	2.97 (1.6–5.47)*	1.11 (0.9–1.36)	<0.001*

CI, Confidence interval; OR, Odds ratio; SEDI, Socio-economic Disadvantage Index

*p<0.05

†Adjusted for Age, sex, ethnicity, diabetes, hypertension, dyslipidemia, cardiovascular disease, and alcohol and smoking status

‡per SD increase in SEDI

^Interaction between individual-level low SES and area-level SEDI.

**Table 4:** Multi-level analysis of socio-economic status with presence of any visual impairment stratified by sub-groups.

Subgroups	Individual level	Area-level
	Low SES	SEDI score‡
	OR (95% CI) †	OR (95% CI) †
**Age group**		
Age group (40–65)	1.98 (1.72–2.27)*	1.11 (1.03–1.19)*
Age group (65–74)	2.32 (1.78–3.02)*	1.04 (0.94–1.14)
Age group (≥75)	1.72 (1.07–2.77)*	1.03 (0.90–1.18)
**Gender**		
Male	1.92 (1.63–2.25)*	1.10 (1.02–1.18)*
Female	2.33 (1.96–2.76)*	1.05 (0.98–1.12)
**Ethnicity**		
Chinese	2.53 (2.09–3.06)*	1.05 (0.97–1.14)
Malay	1.66 (1.31–2.10)*	1.07 (0.96–1.19)
Indian	2.03 (1.68–2.44)*	1.08 (0.99–1.18)
**Individual SES**		
Low SES		1.06 (1.001–1.13)*
No low SES		1.08 (0.99–1.18)
**Areal SEDI (based on DGP)**		
Bukit Batok (100.6)	2.52 (1.84–3.46)*	
Bukit Merah (110.1)	2.40 (1.83–3.14)*	
Bukit Timah (79.8)	3.90 (1.29–11.85)*	
Clementi (100.6)	2.17 (1.62–2.92)*	
Jurong East (99.9)	1.74 (1.24–2.44)*	
Jurong West (101.6)	1.90 (1.54–2.33)*	
Outram (120.1)	4.16 (0.79–22.00)	
Queenstown (106.9)	1.64 (0.91–2.96)	

CI, Confidence interval; OR, Odds ratio; SEDI, Socio-economic Disadvantage Index

*p<0.05

†Adjusted for Age, sex, ethnicity, diabetes, hypertension, dyslipidemia, cardiovascular disease, and alcohol and smoking status

‡Per SD increase in SEDI.

## Discussion

In this large population-based multi-ethnic sample of Asian adults, we found both individual- and area-level SES to be associated with the presence and severity of VI independent of demographic, medical, and life-style risk factors. In addition, we found that the associations between area-level SEDI score and VI to be more pronounced in certain subgroups such as adults aged 40–65 years and males. To our knowledge, this is the first study to use both individual- and area-level disadvantage indices to assess socioeconomic disparities in visual outcomes in Asia. Importantly, our findings show that although Singapore has the third highest life expectancy in the world and a low infant mortality rate (2 per 1,000 live births) in 2013[31]; the association of socioeconomic disadvantage with VI suggests that a similar or worse event may be evident in other developed countries worldwide. The SEDI score created in our study may provide a methodology for the assessment of the impact of area-level SES on VI in other Asian communities.

Previous studies examining the association of disadvantage index with VI in US, Europe, South Africa and Australia have shown inconsistent results[25,32–35]. Neighbourhood SES was found to be associated with low vision[25], late presentation of glaucoma[36] and severity of glaucoma at presentation[37] but few studies have reported no association of SES with VI[33] or presenting VA in those with age-related macular degeneration[38]. Our findings are consistent with the EPIC-Norfolk Eye Study in Europe reporting both individual and area-level disadvantage index to be associated with VI and extends the findings to Asian populations[25]. However, the effects of neighbourhoods are small in comparison with the individual-level effect of being in a low SES group. Several studies have shown area-level socioeconomic disadvantage to be associated with major risk factors of VI including diabetes, and hypertension and adverse health outcomes including depression, CVD and mortality [39–44].

Neighbourhood environment impacts health outcomes through mechanisms such as availability of healthcare services; physical and financial access to health care; infrastructure facilities (for e.g. parks and exercise facilities) that support healthy lifestyle; environmental pollution; and attitude towards health behaviour[2,45,46]. Studies that reported an association between neighbourhood SES and visual outcomes suggested access to care as one of the mediating factors, for example those living in areas with fewer eye care services[35,47]. or those with no insurance coverage[48] were more likely to have adverse visual outcomes.

In Singapore, most areas are well-connected to health care offering vision services and therefore, physical access to care is unlikely to explain socio-economic disparities in vision related outcomes. The Singapore health care financing system comprises of means-tested government subsidies ranging between 20% and 80%; and the balance paid by patients out-of-pocket (for out-patient) or from Medisave (for in-patient)[49–50]. The reason for the socio-economic disparities in VI is therefore not clear in Singapore. Cataracts accounted for the major cause of blindness in this study. An earlier report that showed low SES to be significantly associated with cataract (for out-patient diagnosis) but not with cataract surgery which is readily affordable to most citizens in Singapore through government subsidy and Medisave payments[51,52]. Socio-economic disadvantage has been suggested to influence one’s ability to access refractive error correction[53–54]. As under-corrective refractive error accounted for the majority of VI and low vision in this population, the out-of-pocket costs to correct under-corrective refractive error, an out-patient service, could explain the socioeconomic disparity in VI in this population. Inadequate literacy was found to be associated with VI among Singaporean Malays and those with limited literacy were more likely to be elderly and had lower income[9]. Therefore, poor health literacy and lack of awareness could have contributed to blindness among those with low SES in Singapore. In addition, those in the low SES could have poor dietary habits or poor metabolic profile leading to increased prevalence of major blinding eye diseases such as age-related macular degeneration, or Diabetic retinopathy[55,56]. In the current study, consistent with other studies, females had a higher prevalence of blindness than males[57]. That could possibly be explained by longer life expectancy[58], lesser education[59], greater biological susceptibility to ocular conditions leading to blindness[57], lower prevalence of cataract surgery[52], and poorer visual outcomes following cataract surgery[52] among females in Singapore.

As the need for eye care services such as annual eye examination, refractive correction and cataract surgery in Singapore is expected to be substantially higher in future due to rapid aging of the population, urbanisation and increasing prevalence of diabetes and hypertension, more targeted public health interventions such as providing free eye screening services and glasses and increasing subsidises for cataract eye surgery are needed to reduce socioeconomic disparities in vision health.

The strengths of this study include a large, representative, and population-based design and the use of multi-level mixed effects model to adjust for potential individual confounders. Our study has some limitations though. First, we derived our SEDI score using the socio-economic indices from the 2010 census data and it might not entirely reflect SES of participants at the time since outcome data were collected from 3 different periods. Second, due to the cross-sectional nature of the study design, causal inferences cannot be made, for example, we may not be able to determine if those residing in low SES areas develop VI or those with VI move to low SES areas. Third, findings from this Asian population in Singapore might not be generalizable to other Asian population in the region due to differences in health care systems, prevalence of eye diseases and composition of ethnic groups. Additionally, it should be noted that SEDI scores reflect the disadvantage of areas that individuals reside in, rather than the individuals themselves. Not all individuals who live in an area with high SEDI scores are disadvantaged, and similarly a person who lives in an area with low SEDI score may be disadvantaged. Finally, a large-scale study comprising of a nationally representative population is needed to confirm this socio-economic association with VI in Singapore.

In conclusion, we found an independent positive association between individual and area-level SES with the presence and severity of VI. Our findings, if confirmed in future prospective studies, may have implications for developing targeted public health interventions aiming to reduce the burden of visual loss in those living in low SES areas in addition to individual SES.

## Abbreviations

SES: - Socio-economic status
VI: - Visual impairment
SEDI: - Socio-economic disadvantage index
SEED: - Singapore epidemiology of eye disease
SiMES: - Singapore Malay eye study
SINDI: - Singapore Indian eye study
SCES: - Singapore Chinese eye study
SGD: - Singapore dollars
DGP: - Development guide plan
SD: - Standard deviation
VA: - Visual acuity
CVD: - Cardiovascular disease
PVA: - Presenting visual acuity
